# The Pattern of Complementary Foods in American Infants and Children Aged 0–5 Years Old—A Cross-Sectional Analysis of Data from the NHANES 2011–2014

**DOI:** 10.3390/nu10070827

**Published:** 2018-06-26

**Authors:** Elieke Demmer, Christopher J. Cifelli, Jenny A. Houchins, Victor L. Fulgoni

**Affiliations:** 1 National Dairy Council, 10255 West Higgins Road, Suite 900, Rosemont, IL 60018-5616, USA; eliekedemmer@gmail.com (E.D.); jenny.houchins@dairy.org (J.A.H.); 2 Nutrition Impact, LLC, Battle Creek, MI 49014, USA; VIC3RD@aol.com

**Keywords:** dairy, protein, vegetable, added sugar, calcium, vitamin D, vitamin E

## Abstract

Proper nutrition early in life can influence children’s present and future health. While several authoritative sources provide eating/food recommendations, only a few studies have assessed whether these recommendations are followed. The goal of this paper was to examine food and nutrient intakes on any given day during infancy and early childhood among various ethnicities. Twenty-four-hour dietary recall data of 0–5 years-old children (*n* = 2431) from the National Health and Nutrition Examination Survey (NHANES) 2011–2014 was used to estimate intakes of nutrients and food groups and prevalence of inadequate intake. Data was analyzed separately for various age groups and ethnicities, and differences in means by age and or race/ethnicity were determined by *t*-tests with *p* < 0.05 as significant. The results indicate that intakes of all food groups were expectedly low at 0–11 months, increased with age, and were influenced by race/ethnicity. Mixed dish consumption, which also increased with age, made substantial contributions to children’s food group intakes. However, there was a substantial percentage of the population among all age and race/ethnic groups who did not consume the recommended amounts for each food group and had inadequate intakes of key nutrients, such as calcium, vitamin D, and vitamin E. Non-Hispanic black children consumed less dairy and more protein foods, and a significantly greater proportion of these children had inadequate intakes of calcium and vitamin D compared to their peers. In conclusion, the results from this study suggest that a substantial population of American infants and children from 0 to five years of age did not meet food group recommendations and had inadequate intakes of key nutrients such as calcium, vitamin D, and vitamin E from foods.

## 1. Introduction

Proper nutrition early in life can affect development and help set the stage for future eating habits that can influence nutrition status for a lifetime [1]. As such, the American Academy of Pediatrics (AAP) states that “an important goal of early childhood nutrition is to ensure children’s present and future health by fostering the development of healthy eating behaviors” and that “appropriate feeding behavior is vital in promoting healthy growth and development but also in engendering healthy behavior and habits that can prevent the advent of chronic disease” [2]. While several authoritative sources provide recommendations on what, when, and how much children should be eating [2,3], few studies have assessed whether these recommendations are followed, how these food choices impact nutrient intake, and what, if any, differences exist among race/ethnic groups. Ethnic disparities in dietary patterns are reflective of biological, cultural, and food practice differences that are further modified by social, economic, and environmental factors [4,5]. Given that the 2020 Dietary Guidelines for Americans (DGA) will include the first two years of life for the first time [6,7], it is critical to understand the eating behaviors and patterns of this young population. One specific question posed by the joint United States Department of Agriculture (USDA) and Health and Human Services “Pregnancy and Birth to 24 Months project” is “What is the relationship between complementary feeding and micronutrient status” [8]. 

The AAP recommends human milk as the sole source of nutrients for healthy term infants with iron-fortified formula as the appropriate substitute for human milk [2]. While the AAP supports exclusive breastfeeding for approximately six months, it recognizes that infants may be developmentally ready to accept complementary foods at four to six months of age [2]. Complementary foods are defined as nutrient- and energy-containing solid or semi-solid foods fed to infants in addition to human milk and formula [2]. The introduction of complementary foods prior to four months of age is thought to contribute to later overweight status [9,10,11,12]. While single-grain fortified infant cereal is often the first complementary food introduced, there is no medical evidence that introducing solid foods in any order has any advantage. As such, the AAP recommends that single-ingredient foods from any food group can be introduced at this time [2]. In fact, previous research has shown that after six months of age, infants accept cereals and meats equally well [13]. 

Energy requirements of toddlers are difficult to estimate, and the appropriateness of energy intake is best judged by growth [2]. It has been estimated that between 12–24 months of age, 49–63% of energy comes from table foods [14]. While most American toddlers’ diets contain adequate amounts of protein and carbohydrate, findings from the 2008 Feeding Infants and Toddlers Study (FITS) suggest that nearly 25% of toddlers had intakes of fat below recommended levels [15]. The AAP recommends against fat or cholesterol restrictions for infants younger than two years, when rapid growth and neurologic development requires high energy intakes, unless a concern for obesity or family history of cardiovascular disease exists [16]. 

The goal of this paper was to examine what American infants and children from 0–5 years are eating, what they consume on any given day during infancy and childhood, how these food choices influence nutrient intake, and to determine if differences exist among ethnic groups at this young and important age. 

## 2. Methods

### 2.1. Subjects

Data from National Health and Nutrition Examination Survey (NHANES), a large dietary survey of a nationally representative sample of the U.S. population, was used to assess dietary intakes and introduction of complementary foods to children of ethnic subgroups in the U.S. population [17]. The present analysis combined two NHANES datasets (NHANES 2011–2012 and 2013–2014). Dietary data (24-h recalls provided by parents/caretakers) from children aged five years and younger (*n* = 2431; 1290 from NHANES 2011–2012 and 1141 from 2013–2014) were included (Table 1), which represents over 22 million U.S. children aged 0–5 years [18]. Children with incomplete or unreliable data (as determined by the USDA) were excluded from the analysis (*n* = 507) as were those consuming breast milk (*n* = 260); the latter were excluded so as not to overestimate those with nutrient inadequacy given we do not have data on the amount of breastmilk consumed. All parents/caretakers or proxies provided written informed consent, and the Research Ethics Review Board at the NCHS approved the survey protocol. Analysis of de-identified data from the survey is exempt from the federal regulations for the protection of human research participants. Analysis of restricted data through the NCHS Research Data Center is also approved by the NCHS ERB. This study did not require further institutional review as this was the analysis of secondary data without any personal identifiers.

### 2.2. Estimation of Intake

Dietary intake data were obtained via 24-h dietary recall interviews with caretakers of children using an Automated Multiple-Pass Method [19]. Complementary food intake was assessed using USDA food codes corresponding to What We Eat In America (WWEIA), the dietary intake component of NHANES [20]. Food subgroups and all baby foods (USDA food category starting with 90) were reassigned to broader food group categories (e.g., ‘banana baby food: fruit’ was added to the ‘fruit’ group) [20]. Energy and nutrient intake from foods were determined using respective Food & Nutrient Database for Dietary Studies (FNDDS) for each NHANES cycle [21,22] available from total nutrients intake files. Use of supplements was not included in these analyses. Intake of MyPlate servings [23] was calculated using the Food Patterns Equivalents Databases (FPED) 2011–2012 and 2013–2014 for NHANES 2011–2012 and 2013–2014 dietary data, respectively. The number of MyPlate servings was aggregated over all foods consumed during the 24-h recall to calculate the daily intakes of MyPlate food groups. 

### 2.3. Statistical Analyses

SAS 9.2 (SAS Institute, Cary, NC, USA) and SUDAN 11 were used with NHANES design aspects and sampling weights to ensure nationally representative estimates. Usual nutrient intakes of energy and nutrients were determined using the National Cancer Institute method using both days of dietary intake [24]. The percentage of the population below the Estimated Average Requirement (EAR) was assessed. Amounts of MyPlate food groups consumed and the percent of the population not meeting the age specific recommendations for various food groups [3,25] were also computed. Covariates used for the usual intake models were dietary recall number (1 or 2) and day of recall coded as weekday or weekend (Fri, Sat, and Sun). Separate analyses were conducted for the following age groups in the U.S. population (males and females combined): 0–11 months, 12–23 months, 2–3 years, and 4–5 years; and self-identified ethnic groups (males and females combined): non-Hispanic whites (NH-whites), non-Hispanic blacks (NH-blacks), Hispanics, and Asians (all of which are sampled to be nationally representative). Mean amounts and standard errors (SE) were determined with “Proc Surveymeans” procedure, while for analyses of ethnicity by age, “Proc Surveyreg” procedure was used with age (in months), gender, race/ethnicity and family poverty income ratio included as covariates; differences in means and least square means by age and ethnicity were determined by *t*-tests while differences in percentages below recommendations were assessed with the *Z*-statistic. *p* < 0.05 as deemed significant.

## 3. Results

Reported intakes of various food groups among children analyzed by age are presented in Table 2. Regardless of ethnicity, intake of all food groups among 0–11 months-old children was low. Intake of total dairy increased as children got older, with a maximum average intake of 2.44 cup equivalents among those aged 12–23 months; after 23 months of age, dairy consumption began to decrease. Protein and grain intake increased as children got older. The amount of fruit consumed increased to a little above 1 cup per day when children were 12–23 months-old and then decreased after three years of age. Vegetable consumption increased with age, although the amounts consumed were consistently very low, never getting above 0.7 cups per day. The consumed amounts of solid fats, oils, and added sugars quickly increased as children got older. The largest increase in intake of various food groups (~800% increase in dairy, ~447% increase in protein, ~250% increase in grains and fruits intake) was noted between the ages 0–11 months and 12–23 months. Similarly, intake of solid fat and added sugar increased by more than 500% between age groups 0–11 months and 12–23 years. Mixed dish consumption, which also increased with age, made substantial contributions to children’s food intake. Among 4–5 year-old children, mixed dishes contributed 16% of dairy intake (52% of cheese intake, data not shown), 27% of protein intake, 31% of grains and vegetables intake, and 45% of solid fat intake (Table 2). 

Intakes of food groups among children differed by ethnicity (Table 3). NH-black children ages 12–23 months, 2–3 years, and 4–5 years consumed significantly less dairy than NH-white and Hispanic children. After 1 year of age, dairy consumption was consistently the lowest among NH-black children. However, the prevalence of cheese consumption among NH-black children was similar to NH-white and Hispanic children (data not shown). Asian children consumed more yogurt than the other ethnic groups (data not shown). Older NH-white and Hispanic children (2–3 years and 4–5 years of age) consumed significantly less protein foods than NH-black children. At 4–5 years of age, Asian children consumed significantly less fruit than NH-white children. Intake of added sugars among 4–5-year-old children was more than the American Heart Association’s (AHA’s) recommended daily limit of 6 teaspoons [26] for all ethnicities except for Hispanic children, who consumed significantly less added sugar at this age than their NH-white and NH-black peers (Table 3). Intake of grains, vegetables and solid fat were not significantly different among ethnic groups.

Large percentages of children from all ethnic groups consumed less dairy, protein foods, vegetables, and oil than the recommendations for their respective age groups (Table 4). The percent of children with intakes below recommendations increased with age for dairy, fruit, and vegetables. The proportion of children with intakes below the dietary recommendations varied by ethnicity. Across all age groups, NH-black children consistently had the greatest percentage of children not meeting the dairy food group recommendations. In contrast, this ethnic group also had the lowest prevalence of inadequate protein foods and oil intake. However, in general there were few significant ethnic differences in the percent of the population who had intakes below the recommendations for fruits or vegetables (Table 4). 

Intakes of energy and macronutrients generally increased with age (Table 5). With a few exceptions, there were no differences in energy and macronutrient intakes when analyzed by ethnicity. NH-black children aged 12–23 months consumed significantly more energy (8%) than their Hispanic peers as well as significantly more carbohydrate (12%) than their Hispanic and NH-white peers. Hispanic children aged 2–3 years consumed significantly more protein than 2–3-years-old NH-white (8%) and NH-black (10%) children. Four to five-year-old NH-black children also consumed significantly more fat (14%) than 4–5-year-old Asian children (Table 5).

Table 6 shows the proportion of children who had inadequate intakes (i.e., intakes below EAR) for various micronutrients from foods by age and ethnicity. The proportion of children with inadequate micronutrient intakes was generally low (less than 10% of the population) except for calcium (NH-black aged 2–3 years, and all ethnicities aged 4–5 years), Vitamin D (all ages and ethnicities), and vitamin E (all ages and ethnicities). A significantly higher proportion of 4–5 years-old NH-black children had inadequate intakes of calcium compared to NH-white and Hispanic children. Vitamin D inadequacy was generally higher in NH-black children as compared to Hispanic and NH-white children, while the opposite was true for vitamin E (Table 6). 

## 4. Discussion

This report, using the nationally representative NHANES 2011–2012 & 2013–2014 dietary data, explored the consumption patterns of American infants and children by age and ethnicity and compared the current intakes with the age appropriate dietary recommendations. The results indicate that intakes of all food groups were low among 0–11 month-old children. While there was an increase in overall food consumption with age, there was a substantial percentage of the population among all age and ethnic groups who did not consume the recommended amounts for each food group and had inadequate intakes of key nutrients, such as calcium, vitamin D, and vitamin E from foods. 

The results show that the intake of various food groups was low during the first year of life and increased gradually with age, suggesting increased intake of complementary foods. Complementary feeding generally starts when human milk or formula is no longer nutritionally sufficient. It is a major step in the development of eating behavior and directly affects the infant’s growth and health [27,28,29]. Single-grain fortified infant cereals are often the first complementary food introduced and our data supports this assertion by showing that the intake of grain foods increased from 0.85 oz. eq. during 0–11 months to more than three oz. eq. at 12–23 months of age. Data from the Infant Feeding Practices Study II indicated that by four months of age, 40% of infants had consumed cereal, 17% had consumed fruits or vegetables [30], and more than 40% of mothers reported having introduced solid foods before their infant was four months old [31]. Another study also reported that 62% of parents introduced solid foods within the recommended 4–6 months age window while about 19% introduced solid foods prior to 4 months of age and about 19% started at 7 months of age or later [32]. Early and late introduction of solid foods (outside the 4–6 months window) is not optimal for infant health and nutrition as it may lead to increased risk for the development of chronic diseases such as islet autoimmunity (the pre-clinical condition leading to type 1 diabetes), obesity, adult-onset celiac disease, and eczema [33,34,35].

The AAP states that there is no reason to introduce whole food groups one after the other, instead, they recommend a gradual introduction of foods from all groups as soon as the infant is developmentally ready. The goal of complementary feeding is to eventually have the child eat the same well-balanced, nutritious mixed diet of ‘family foods’. With that in mind, the AAP recommends that by 7–8 months of age, infants should be consuming foods from all food groups, including a variety of foods each day that may include: human milk/formula, meats, cereal, vegetables, fruits, eggs, and fish [2]. While children may not accept new foods right away, parents should be encouraged to offer foods multiple times (>10 exposures) for infants and toddlers to become accepting of new foods [36].

Our data show that the intakes of added sugar and fat increased with age, and mixed dishes contributed significant amounts to food groups. Contribution of mixed dishes increased at age 12–23 months and made up 25% of total grains and protein intake while more than 50% of fats came from mixed dishes. As children got older, the contributions of mixed dishes increased for each food group. Total dairy intake decreased after age 12–23 months, but contribution of mixed dishes to dairy intake continued to increase with age. At 4–5 years, mixed dishes contributed about 16% of total dairy but contributed 52% of cheese intake (data not presented), likely due to pizza consumption. Interestingly, mixed dishes also contributed about 30% of grain, protein, and vegetable intake (other components of pizza) for 4–5 years old children. Young children have high nutrient needs and relatively low energy requirements, leaving little room for added sugar- and fat-dense foods [16]. The 2015–2020 DGA recommend that no more than 10% of daily calories come from added sugars [3]. While the AAP has recognized that limited amounts of added sugars may be used to increase palatability of nutritious food options [37], the AHA recommends that children younger than two years of age should avoid added sugar all together. Additionally, the AHA states that if added sugar is consumed after two years of age, children should consume no more than 6 teaspoons per day due to the association between added sugar and increased CVD risk [26]. Our data showed that, regardless of ethnicity, the mean intake of added sugar among 4–5 years-old slightly exceeded the AHA recommendation. Given the association between added sugar consumption and increased energy intake and adiposity [26], our data suggest that the persistent over-consumption of added sugars beginning early in life could partially contribute to childhood obesity. Ethnic differences did exist among the added sugar consumption rates for 4–5 years-old children. Hispanic children consumed 27–39% less sugar than their NH-black and NH-white peers. These results align with a recent study where NH-white children were reported to have a higher mean intake of added sugars compared to Mexican American children [38]. In a small population study, NH-black children preferred significantly higher concentrations of sucrose when compared to NH-white children, which the authors attributed to differences in demographic factors, cultural practices, and genetic variation [39].

Our data also show that while about 19% of 12–23-month old children did not meet recommendations for dairy, the prevalence of inadequate dairy consumption increased with age; over 70% of children aged 4–5 years and over 60% children 2–5 years consumed less dairy than recommended and this represents over five million children in each of these age groups with dairy intakes below recommended levels. NH-black children 1–5 years of age consistently consumed about 30% less dairy than their peers of other ethnicities. Similar findings among older children have been reported previously [40,41]. In a small cross-sectional study, NH-black preschool children were reported to consume less dairy that their Hispanic peers, and a greater proportion of NH-black children across all age groups, compared to their peers of other ethnicities, did not meet the dairy food group recommendations [42]. This finding may be explained by the perceived genetic predisposition of the NH-black population toward lactose intolerance, as well as cultural beliefs and perceptions about the consumption of dairy products [43]. Since a significantly greater proportion of 2–3 and 4–5 years-old NH-black children had inadequate intakes of calcium and vitamin D compared to their peers of other ethnicities, consuming adequate foods from the dairy food group, which provides both of these essential nutrients, could be a simple and affordable solution [44].

Our data showed that protein foods were consistently under-consumed; over 70% of all 12–23 months-old children did not meet the protein food group recommendations (over 2.5 million children). Older NH-black children (2–3 and 4–5 years-old) consumed about 30% more protein foods then their NH-white and Hispanic peers, which is consistent with previous studies where NH-black children consumed more meat than Hispanic children [42]. In an earlier analysis of NHANES 2001–2004, a smaller proportion of NH-white children age 2–16 years met the meat and beans recommendations compared to their NH-black and Mexican American counterparts [40]. Meat is the main dietary source of iron and zinc; however, our results did not find any ethnic difference in prevalence of inadequate intake of these minerals. 

The use of a cross-sectional study design was a major limitation of this study, preventing any determination of cause and effect. Additionally, the estimates of dietary intakes among young children were based on self-reported 24-h dietary recalls, which primary rely on the memory of parents/caregivers and as such are subject to reporting bias and may not fully account for day-to-day variations. Further, supplement intake was not included in our analysis, which would impact nutrient adequacy. Major strengths of this study included the use of a large nationally representative sample achieved through combining several sets of NHANES data, including for the first time nationally representative data for the Asian population.

## 5. Conclusions

In conclusion, the results from this study suggest that a substantial population of American infants and children from 0–5 years of age and various ethnic groups did not meet food group recommendations and had inadequate intakes of key nutrients such as calcium, vitamin D, and vitamin E from foods. Consumption of more nutrient dense foods (e.g., milk, fortified cereals, high omega-3 fish, fruits, etc.) should be encouraged to increase intakes of these and other nutrients; if this cannot be accomplished then an addition of dietary supplements should be considered. Additionally, our data showed that ethnic disparities exist for food intake among the very young American population, with NH-black children particularly standing out as the group who consistently consumed the least amount of dairy from 1–5 years of age. 

## Figures and Tables

**Table 1:** Sample sizes by age and race/ethnicity, NHANES 2011–2014.

	0–11 Months	12–23 Months	2–3 Years	4–5 Years
All	526	394	836	675
Non-Hispanic White	145	91	197	133
Non-Hispanic Black	122	85	210	156
Hispanic	158	131	244	223
Asian	25	33	78	72

**Table 2:** Amount of food consumed per food group, NHANES 2011–2014.

	0–11 Months (Mean ± SE)	12–23 Months (Mean ± SE)	2–3 Years (Mean ± SE)	4–5 Years (Mean ± SE)
Total Dairy ^1^ (cups eq.)	0.27 ± 0.03	2.4 ± 0.10	2.2 ± 0.08	2.0 ± 0.09
Mixed dishes	0.02 ± 0.005	0.18 ± 0.03	0.23 ± 0.02	0.32 ± 0.02
Total Protein ^2^ (oz. eq.)	0.36 ± 0.04	2.0 ± 0.11	2.7 ± 0.11	3.0 ± 0.18
Mixed dishes	0.09 ± 0.01	0.49 ± 0.04	0.59 ± 0.04	0.81 ± 0.08
Total Grains ^3^ (oz. eq.)	0.85 ± 0.07	3.1 ± 0.13	4.5 ± 0.14	5.5 ± 0.15
Mixed dishes	0.19 ± 0.03	0.98 ± 0.10	1.3 ± 0.09	1.7 ± 0.09
Total Fruit ^4^ (cups eq.)	0.34 ± 0.03	1.2 ± 0.06	1.4 ± 0.07	1.2 ± 0.07
Mixed dishes	0.01 ± 0.003	0.001 ± 0.001	0.001 ± 0.0004	0.001 ± 0.001
Total Vegetables ^5^ (cups eq.)	0.26 ± 0.02	0.47 ± 0.04	0.56 ± 0.03	0.66 ± 0.05
Mixed dishes	0.04 ± 0.01	0.18 ± 0.02	0.17 ± 0.01	0.21 ± 0.02
Solid Fats ^6^ (g)	1.1 ± 0.15	8.3 ± 0.89	13.0 ± 0.77	17.0 ± 0.68
Mixed dishes	0.67 ± 0.12	4.4 ± 0.52	6.5 ± 0.49	7.8 ± 0.47
Oils ^7^ (g)	0.74 ± 0.15	4.5 ± 0.28	7.0 ± 0.33	8.9 ± 0.50
Mixed dishes	0.43 ± 0.08	1.8 ± 0.17	2.1 ± 0.13	2.8 ± 0.20
Added Sugars ^8^ (tsp eq.)	0.33 ± 0.06	2.6 ± 0.35	4.3 ± 0.24	6.2 ± 0.30
Mixed dishes	0.02 ± 0.01	0.12 ± 0.02	0.26 ± 0.02	0.40 ± 0.05

SE, Standard Error; eq, equivalents. ^1^ Total Dairy include: Milk, flavored milk, dairy drinks and substitutes, total cheese, total yogurt, dairy from mixed dishes, dairy from snacks and sweets; ^2^ Protein include: meats, poultry, seafood, eggs, cured meats/poultry, plant-based protein foods, baby food meat, protein from mixed dishes, protein from snacks and sweets; ^3^ Grains include (whole and refined): cooked grains, breads, rolls, tortillas, quick breads and bread products, ready-to-eat cereals, cooked cereals, baby food cereals, baby food grains, grains from mixed dishes, grains from snacks and sweets; ^4^ Total Fruits include: whole fruit, 100% fruit juice, baby food fruit, fruit from mixed dishes, fruit from snacks and sweets; ^5^ Vegetables include: vegetables excluding potatoes and legumes, white potatoes, 100% vegetables juice, baby food vegetables, vegetables from mixed dishes, vegetables from snacks and sweets; ^6^ Solid fats include: butter and animal fats, margarine, cream cheese, sour cream, whipped cream, cream and cream substitutes, mayonnaise, salad dressings and vegetable oils, solid fat from mixed dishes, solid fat from snacks and sweets; ^7^ Oils include: whipped cream, cream and cream substitutes, mayonnaise, salad dressings and vegetable oils, oils from mixed dishes, oils from snacks and sweets; ^8^ Added Sugars include: sugars and honey, sugar substitutes, jams, syrups, toppings, added sugars from mixed dishes, added sugar from snacks and sweets.

**Table 3:** Amount of food consumed per food group by age and ethnicity, NHANES 2011–2014.

	0–11 Months LSM ± SE	12–23 Months LSM ± SE	2–3 Years LSM ± SE	4–5 Years LSM ± SE
Total dairy ^1^ (cup eq.)				
Non-Hispanic white	0.38 ± 0.05 ^a^	2.6 ± 0.21 ^b^	2.2 ± 0.14 ^b^	2.1 ± 0.19 ^b^
Non-Hispanic black	0.22 ± 0.06 ^ab^	1.8 ± 0.16 ^a^	1.6 ± 0.14 ^a^	1.5 ± 0.11 ^a^
Hispanic	0.16 ± 0.05 ^b^	2.5 ± 0.11 ^b^	2.4 ± 0.13 ^b^	2.2 ± 0.12 ^b^
Asian	0.15 ± 0.10 ^b^	2.3 ± 0.32 ^ab^	2.4 ± 0.32 ^b^	2.1 ± 0.21 ^b^
Total Protein ^2^ (oz. eq.)				
Non-Hispanic white	0.45 ± 0.06 ^a^	2.0 ± 0.20	2.5 ± 0.16 ^a^	2.8 ± 0.29 ^a^
Non-Hispanic black	0.44 ± 0.14 ^ab^	2.0 ± 0.28	3.3 ± 0.27 ^bc^	3.9 ± 0.32 ^b^
Hispanic	0.21 ± 0.04 ^b^	2.0 ± 0.13	2.6 ± 0.14 ^a^	2.8 ± 0.19 ^a^
Asian	0.38 ± 0.17 ^ab^	1.5 ± 0.27	2.7 ± 0.49 ^ac^	2.5 ± 0.29 ^a^
Total grain ^3^ (oz. eq.)				
Non-Hispanic white	0.85 ± 0.10	3.2 ± 0.19	4.2 ± 0.14	5.3 ± 0.26
Non-Hispanic black	0.89 ± 0.15	3.3 ± 0.17	4.6 ± 0.22	5.5 ± 0.30
Hispanic	0.95 ± 0.11	2.9 ± 0.26	4.7 ± 0.28	5.6 ± 0.23
Asian	0.68 ± 0.20	3.3 ± 0.41	5.1 ± 0.54	4.8 ± 0.49
Total fruits ^4^ (cup eq.)				
Non-Hispanic white	0.38 ± 0.04 ^a^	1.2 ± 0.13	1.4 ± 0.12	1.7 ± 0.10 ^a^
Non-Hispanic black	0.25 ± 0.04 ^bc^	1.4 ± 0.15	1.4 ± 0.14	1.7 ± 0.13 ^ac^
Hispanic	0.34 ± 0.05 ^ab^	1.2 ± 0.09	1.3 ± 0.11	1.3 ± 0.10 ^a^
Asian	0.17 ± 0.06 ^c^	1.0 ± 0.17	1.4 ± 0.19	0.85 ± 0.14 ^bc^
Total vegetables ^5^ (cup eq.)				
Non-Hispanic white	0.27 ± 0.02	0.45 ± 0.06	0.51 ± 0.04	0.67 ± 0.08
Non-Hispanic black	0.23 ± 0.04	0.48 ± 0.06	0.56 ± 0.04	0.76 ± 0.07
Hispanic	0.28 ± 0.03	0.48 ± 0.04	0.64 ± 0.06	0.64 ± 0.05
Asian	0.26 ± 0.11	0.56 ± 0.12	0.52 ± 0.08	0.68 ± 0.08
Solid fats ^6^ (g)				
Non-Hispanic white	1.2 ± 0.26	8.7 ± 1.51	12.4 ± 1.3	17.8 ± 0.9
Non-Hispanic black	0.96 ± 0.19	8.8 ± 1.31	14.5 ± 0.9	17.4 ± 1.5
Hispanic	0.88 ± 0.17	7.7 ± 1.10	14.4 ± 0.8	17.4 ± 1.1
Asian	1.2 ± 0.82	5.1 ± 1.33	12.6 ± 1.5	15.4 ± 2.6
Oils ^7^ (g)				
Non-Hispanic white	0.82 ± 0.26	4.2 ± 0.43	6.1 ± 0.37 ^a^	8.3 ± 0.79 ^ab^
Non-Hispanic black	0.60 ± 0.21	6.0 ± 0.93	8.3 ± 0.67 ^bc^	11 ± 1.1 ^b^
Hispanic	0.85 ± 0.16	4.6 ± 0.50	7.9 ± 0.75 ^bc^	8.1 ± 0.68 ^a^
Asian	0.91 ± 0.47	4.0 ± 0.90	7.7 ± 1.51 ^ac^	8.6 ± 1.09 ^ab^
Added sugars ^8^ (tsp eq.)				
Non-Hispanic white	0.31 ± 0.08 ^ab^	2.8 ± 0.77	4.5 ± 0.36	7.4 ± 0.51 ^bc^
Non-Hispanic black	0.27 ± 0.09 ^ab^	2.1 ± 0.52	4.8 ± 0.44	6.1 ± 0.43 ^bc^
Hispanic	0.50 ± 0.16 ^a^	2.4 ± 0.34	4.1 ± 0.33	4.5 ± 0.26 ^a^
Asian	0.07 ± 0.11 ^b^	1.5 ± 0.60	4.1 ± 0.56	7.0 ± 2.27 ^ac^

^a,b,c^ Least square means with different superscripts are significantly different by race/ethnicity within age groups, *p* < 0.05. LSM, Least Squares Mean; SE, Standard Error; eq, equivalents; ^1^ Total Dairy include: Milk, flavored milk, dairy drinks and substitutes, total cheese, total yogurt, dairy from mixed dishes, dairy from snacks and sweets; ^2^ Protein include: meats, poultry, seafood, eggs, cured meats/poultry, plant-based protein foods, baby food meat, protein from mixed dishes, protein from snacks and sweets; ^3^ Grains include (whole and refined): cooked grains, breads, rolls, tortillas, quick breads and bread products, ready-to-eat cereals, cooked cereals, baby food cereals, baby food grains, grains from mixed dishes, grains from snacks and sweets; ^4^ Total Fruits include: whole fruit, 100% fruit juice, baby food fruit, fruit from mixed dishes, fruit from snacks and sweets; ^5^ Vegetables include: vegetables excluding potatoes and legumes, white potatoes, 100% vegetables juice, baby food vegetables, vegetables from mixed dishes, vegetables from snacks and sweets; ^6^ Solid fats include: butter and animal fats, margarine, cream cheese, sour cream, whipped cream, cream and cream substitutes, mayonnaise, salad dressings and vegetable oils, solid fat from mixed dishes, solid fat from snacks and sweets; ^7^ Oils include: whipped cream, cream and cream substitutes, mayonnaise, salad dressings and vegetable oils, oils from mixed dishes, oils from snacks and sweets; ^8^ Added Sugars include: sugars and honey, sugar substitutes, jams, syrups, toppings, added sugars from mixed dishes, added sugar from snacks and sweets.

**Table 4:** Percent of children below the recommended intake of food groups by age and ethnicity, NHANES 2011–2014.

	12–23 Months * Mean ± SE	2–3 Years ** Mean ± SE	4–5 Years ** Mean ± SE
Total dairy ^1^ Recommended, (cup eq.)	1.57	2	2.5
All (%)	18.8 ± 2.9 ^x^	44.3 ± 2.7 ^y^	71.9 ± 3.6 ^z^
Hispanic	13 ± 3.2 ^a^	34.2 ± 4.4 ^a^	63.5 ± 4.9 ^a^
Non-Hispanic white	15.9 ± 5.3 ^a^	39.4 ± 4.3 ^a^	69.7 ± 6.9 ^ab^
Non-Hispanic black	36.9 ± 5.5 ^b^	70.4 ± 4.5 ^b^	83.7 ± 3.1 ^b^
Asian	20.3 ± 7.7 ^ab^	49.5 ± 7.2 ^a^	80.7 ± 5.0 ^b^
Total Protein ^2^ Recommended, (oz. eq.)	2.1	2	4
All (%)	73.1 ± 3.6 ^x^	41.3 ±3.5 ^y^	93.9 ± 2.2 ^z^
Hispanic	70.3 ± 6.6 ^a^	31.8 ± 9.2 ^a^	91.2 ± 3.1 ^ab^
Non-Hispanic white	80.1 ± 6.0 ^a^	53.5 ± 5.6 ^b^	97.1 ± 2.5 ^a^
Non-Hispanic black	54.2 ± 9.3 ^b^	16.3 ± 5.1 ^a^	79.5 ± 5.5 ^b^
Asian	76.3 ± 10.2 ^ab^	40.8 ± 15.6 ^ab^	98.4 ± 2.9 ^a^
Total grain ^3^ Recommended, (oz. eq.)	1.57	3	5
All (%)	3.5 ± 1.2 ^x^	11.2 ± 2.4 ^y^	38.3 ± 2.7 ^z^
Hispanic	8.8 ± 2.8 ^a^	17.0 ± 3.3 ^a^	40.9 ± 3.8
Non-Hispanic white	2.3 ± 1.4 ^b^	10.3 ± 3.1 ^a^	39.8 ± 4.8
Non-Hispanic black	3.0 ± 1.6 ^ab^	14.7 ± 4.1 ^a^	39.3 ± 5.7
Asian	0.02 ± 0.5 ^b^	0.01 ± 2.1 ^b^	8.9 ± 21.3
Total fruits ^4^ Recommended, (cup eq.)	0.5	1	1
All (%)	6.1 ± 1.3 ^x^	34.1 ± 3.4 ^y^	38.3 ± 3.0 ^y^
Hispanic	5.4 ± 2.0	34.0 ± 4.7	33.1 ± 5.3
Non-Hispanic white	6.7 ± 2.6	35.3 ± 6.2	39.8± 5.3
Non-Hispanic black	2.7 ± 1.9	33.3 ± 6.0	41.9 ± 5.7
Asian	11.3 ± 5.7	27.2 ± 7.9	39.7 ± 12.1
Total vegetables ^5^ Recommended, (cup eq.)	0.45	1	1.5
All (%)	45.4 ± 6.6 ^x^	89.6 ± 1.8 ^y^	98.1 ± 1.1 ^z^
Hispanic	39.0 ± 7.6	79.1 ± 4.7 ^a^	98.1 ± 1.0
Non-Hispanic white	48.5 ± 12.5	92.8 ± 3.9 ^b^	98.6 ± 1.9
Non-Hispanic black	42.5 ± 12.3	90.5 ± 3.6 ^ab^	98.3 ± 1.3
Asian	45.6 ± 10.9	89.2 ± 4.2 ^ab^	97.0 ± 1.9
Oils ^6^ Recommended, (tsp)	-	4	4
All (%)	-	81.2 ± 2.7 ^x^	66.5 ± 4.2 ^y^
Hispanic	-	82.5 ± 4.4 ^a^	79.6 ± 5.0 ^a^
Non-Hispanic white	-	85.0 ± 3.8 ^a^	68.0 ± 6.3 ^a^
Non-Hispanic black	-	65.7 ± 6.4 ^b^	43.2 ± 6.7 ^b^
Asian	-	79.0 ± 8.1 ^ab^	65.2 ± 9.7 ^ab^

Weighted populations are 3,700,568, 8,374,256, and 7,928,277 for 12–13 month, 2–3 and 4–5 year groups, respectively. Means with different superscripts are significantly different by age (^x,y,z^) and by race/ethnicity within age groups (^a,b^), *p* < 0.05. SE, Standard Error; eq, equivalents. ^1^ Total Dairy include: Milk, flavored milk, dairy drinks and substitutes, total cheese, total yogurt, dairy from mixed dishes, dairy from snacks and sweets. For 12–23 months old children this food group only includes milk; ^2^ Protein include: meats, poultry, seafood, eggs, cured meats/poultry, plant-based protein foods, baby food meat, protein from mixed dishes, protein from snacks and sweets. For 12–23 months old children this food group only includes beef, poultry, and seafood; ^3^ Grains include (whole and refined): cooked grains, breads, rolls, tortillas, quick breads and bread products, ready-to-eat cereals, cooked cereals, baby food cereals, baby food grains, grains from mixed dishes, grains from snacks and sweets; ^4^ Total Fruits include: whole fruit, 100% fruit juice, baby food fruit, fruit from mixed dishes, fruit from snacks and sweets; ^5^ Vegetables include: 100% vegetables juice, baby food vegetables, vegetables from mixed dishes, vegetables from snacks and sweets. For 12–23 months old children, this food group (vegetables) excludes legumes; ^6^ Oils include: whipped cream, cream and cream substitutes, mayonnaise, salad dressings and vegetable oils, oils from mixed dishes, oils from snacks and sweets; * Recommended amounts of food groups for 12–23 months developed from [22]; ** Recommended amounts of food groups for 2–3 and 4–5 years were derived from [3], translated to serving sizes on www.choosemyplate.gov.

**Table 5:** Usual intake of total energy and macronutrient intake in children by age and ethnicity, NHANES 2011–2014.

	0–11 Months (Mean ± SE)	12–23 Months (Mean ± SE)	2–3 Years (Mean ± SE)	4–5 Years (Mean ± SE)
Energy (kcal)				
All	805 ± 16 ^w^	1253 ± 31 ^x^	1461 ± 19 ^y^	1599 ± 31 ^z^
Hispanic	800 ± 18	1238 ± 29 ^a^	1503 ± 44	1587 ± 42
Non-Hispanic white	803 ± 21	1251 ± 48 ^ab^	1431 ± 31	1599 ± 45
Non-Hispanic black	815 ± 44	1342 ± 38 ^b^	1499 ± 48	1687 ± 60
Asian	803 ± 45	1179 ± 74 ^ab^	1470 ± 57	1538 ± 43
Carbohydrate (g)				
All	102 ± 3 ^w^	164 ± 4 ^x^	199 ± 4 ^y^	220 ± 4 ^z^
Hispanic	102 ± 3	161 ± 5 ^a^	201 ± 6	216 ± 6
Non-Hispanic white	103 ± 4	162 ± 7 ^a^	195 ± 6	221 ± 5
Non-Hispanic black	102 ± 7	181 ± 6 ^b^	203 ± 6	228 ± 7
Asian	102 ± 7	155 ± 12 ^ab^	203 ± 9	211 ± 8
Protein (g)				
All	20.4 ± 0.6 ^w^	47.2 ± 1.1 ^x^	52.9 ± 0.8 ^y^	55.8 ± 1.4 ^y^
Hispanic	20.2 ± 0.8	47.7 ± 1.1	56.1 ± 1.6 ^a^	58.0 ± 1.7
Non-Hispanic white	20.4 ± 0.8	46.6 ± 1.9	51.7 ± 1.0 ^b^	54.9 ± 1.9
Non-Hispanic black	19.8 ± 1.4	46.7 ± 1.4	51.4 ± 1.8 ^b^	56.6 ± 2.4
Asian	20.2 ± 1.5	47.1 ± 3.1	53.9 ± 2.8 ^ab^	54.1 ± 2.1
Total fat (g)				
All	36.5 ± 0.7 ^w^	47.3 ± 1.5 ^x^	52.6 ± 0.9 ^y^	57.4 ± 1.6 ^z^
Hispanic	35.7 ± 0.9	46.2 ± 1.1	54.0 ± 2.0	56.5 ± 1.8 ^ab^
Non-Hispanic white	36.3 ± 0.9	48.8 ± 2.7	51.8 ± 1.5	57.3 ± 2.3 ^ab^
Non-Hispanic black	38.4 ± 1.5	48.4 ± 2.0	55.0 ± 2.4	63.1 ± 3.3 ^a^
Asian	35.6 ± 1.8	42.6 ± 2.6	50.7 ± 2.4	55.3 ± 2.2 ^b^

Means with different superscripts are significantly different by age (^w,x,y,z^) and by race/ethnicity within age groups (^a,b^), *p* < 0.05. SE, Standard Error; g, grams.

**Table 6:** Percent of children below the Estimated Average Requirement (EAR) by age and ethnicity, NHANES 2011–2014.

	12–23 Months (Mean ± SE)	2–3 Years (Mean ± SE)	4–5 Years (Mean ± SE)
Calcium (mg), EAR	500	500	800
All (%)	2.3 ± 0.75 ^x^	3.2 ± 0.66 ^x^	33 ± 3.1 ^y^
Hispanic	1.5 ± 0.87 ^ac^	<1 ^a^	22 ± 5.0 ^a^
Non-Hispanic white	1.3 ± 0.88 ^ac^	2.5 ± 0.85 ^a^	32 ± 4.6 ^ac^
Non-Hispanic black	6.7 ± 2.62 ^a^	12.0 ± 3.5 ^b^	49 ± 5.9 ^b^
Asian	1.0 ± 1.18 ^bc^	2.04 ± 1.34 ^a^	40 ± 6.9 ^bc^
Iron (mg), EAR	3.0	3.0	4.1
All (%)	1.9 ± 0.55 ^xy^	<1 ^x^	2.3 ± 0.36 ^y^
Hispanic	3.5 ± 1.00 ^a^	<1 ^a^	2.3 ± 0.52
Non-Hispanic white	1.6 ± 0.62 ^ab^	1.2 ± 0.36 ^a^	2.6 ± 0.57
Non-Hispanic black	<1 ^ab^	<1 ^ab^	1.5 ± 0.67
Asian	<1 ^b^	<1 ^b^	1.04 ± 0.96
Magnesium (mg), EAR	65	65	110
All (%)	<1 ^x^	<1 ^x^	1.18 ± 0.44 ^y^
Hispanic	<1	<1	<1
Non-Hispanic white	<1	<1	<1
Non-Hispanic black	<1	<1	<1
Asian	<1	<1	<1
Phosphorus (mg), EAR	380	380	405
All (%)	<1	<1	<1
Hispanic	<1	<1	<1
Non-Hispanic white	<1	<1	<1
Non-Hispanic black	<1	<1	<1
Asian	<1	<1	<1
Vitamin A, RAE (mcg), EAR	210	210	275
All (%)	<1 ^x^	<1 ^x^	3.4 ± 1.09 ^y^
Hispanic	<1	<1	1.3 ± 0.87
Non-Hispanic white	<1	<1	3.0 ± 1.43
Non-Hispanic black	2.2 ± 1.4	2.2 ± 1.7	8.1 ± 4.52
Asian	<1	<1	7.5 ± 3.76
Vitamin C (mg), EAR	13	13	22
All (%)	<1 ^x^	<1 ^x^	2.2 ± 0.70 ^y^
Hispanic	<1	<1	0.71 ± 0.38
Non-Hispanic white	<1	<1	3.2 ± 1.7
Non-Hispanic black	<1	<1	0.54 ± 0.43
Asian	2.6 ± 1.5	<1	6.5 ± 3.8
Vitamin D (D2 + D3) (mcg), EAR	10	10	10
All (%)	78 ± 3.4 ^x^	90 ± 1.7 ^y^	94 ± 1.6 ^y^
Hispanic	74 ± 3.4 ^a^	84 ± 2.8 ^a^	90 ± 1.8 ^a^
Non-Hispanic white	76 ± 5.3 ^a^	91 ± 2.4 ^a^	94 ± 2.9 ^ac^
Non-Hispanic black	92 ± 4.3 ^b^	97 ± 1.7 ^b^	98 ± 1.0 ^bc^
Asian	76 ± 7.9 ^ab^	91 ± 5.1 ^ab^	97 ± 2.3 ^bc^
Vitamin E (α-tocopherol, mg), EAR	5	5	6
All (%)	66 ± 4.5 ^x^	48 ± 3.1 ^y^	59 ± 3.6 ^x^
Hispanic	79 ± 4.2 ^a^	53 ± 6.1	72 ± 4.2 ^a^
Non-Hispanic white	61 ± 8.15 ^bc^	51 ± 5.1	59 ± 5.9 ^ac^
Non-Hispanic black	55 ± 7.5 ^b^	46 ± 5.2	43 ± 4.7 ^b^
Asian	81 ± 9.7 ^ac^	40 ± 8.2	52 ± 8.3 ^bc^
Zinc (mg), EAR	2.5	2.5	4.0
All (%)	<1	<1	<1
Hispanic	<1	<1	<1
Non-Hispanic white	<1	<1	<1
Non-Hispanic black	<1	<1	<1
Asian	<1	<1	<1

Weighted populations are 700,568, 8,374,256, and 7,928,277 for 12–13 month, 2–3 and 4–5 year groups, respectively. Nutrient intakes were determined from food intake only. Means with different superscripts are significantly different by age (^x,y^) and by race/ethnicity within age groups (^a,b,c^), *p* < 0.05. EAR, Estimated Average Requirement; SE, Standard Error; mg, milligrams; mcg, micrograms.
